# Local Secretory Trafficking Pathways in Neurons and the Role of Dendritic Golgi Outposts in Different Cell Models

**DOI:** 10.3389/fnmol.2020.597391

**Published:** 2020-11-26

**Authors:** Jingqi Wang, Lou Fourriere, Paul A. Gleeson

**Affiliations:** Department of Biochemistry and Molecular Biology, Bio21 Molecular Science and Biotechnology Institute, The University of Melbourne, Melbourne, VIC, Australia

**Keywords:** neuronal dendrites, Golgi morphology, Golgi stacks, Golgi outposts, membrane trafficking

## Abstract

A fundamental characteristic of neurons is the relationship between the architecture of the polarized neuron and synaptic transmission between neurons. Intracellular membrane trafficking is paramount to establish and maintain neuronal structure; perturbation in trafficking results in defects in neurodevelopment and neurological disorders. Given the physical distance from the cell body to the distal sites of the axon and dendrites, transport of newly synthesized membrane proteins from the central cell body to their functional destination at remote, distal sites represents a conundrum. With the identification of secretory organelles in dendrites, including endoplasmic reticulum (ER) and Golgi outposts (GOs), recent studies have proposed local protein synthesis and trafficking distinct from the conventional anterograde transport pathways of the cell body. A variety of different model organisms, including *Drosophila*, zebrafish, and rodents, have been used to probe the organization and function of the local neuronal secretory network. Here, we review the evidence for local secretory trafficking pathways in dendrites in a variety of cell-based neuronal systems and discuss both the similarities and differences in the organization and role of the local secretory organelles, especially the GOs. In addition, we identify the gaps in the current knowledge and the potential advances using human induced pluripotent stem cells (iPSCs) in defining local membrane protein trafficking in human neurons and in understanding the molecular basis of neurological diseases.

## Introduction

Neurons are highly polarized cells with a central cell body (soma) and multiple branched dendrite extensions and a long axon. Spatial–temporal regulation of intracellular membrane trafficking is essential to establish the architecture of the neural plasma membrane domains; a process which is critical to drive the development, maintenance and plasticity of the neural circuit. The unique cellular architecture and complex geometry of neurons imposes enormous challenges for efficient protein trafficking. How axonal and dendritic proteins navigate through this complex, compartmentalized neuronal structure is a critical issue in neuronal cell biology.

In the secretory pathway of eukaryotic cells, nascent proteins are modified and matured through a series of membrane-enclosed organelles before they reach their functional destination. Correctly folded nascent (glyco) proteins are transported from the ER through the intermediate compartment (ERGIC) to the Golgi apparatus where a variety of post-translational modifications take place; the mature proteins are then sorted at the TGN into transport vesicles (Viotti, 2016). In neurons, the physical distance between the cell body of the neuron and the functional destination for membrane proteins poses a major challenge for rapid and precise protein cargo delivery, especially to synapses at distal locations (Kang and Schuman, 1996).

A solution to this quandary has been the recognition over the past two decades of a distinct secretory system at sites remote from the central secretory machinery in the cell body. Local secretory transport has been reported for both the dendrites and axons of neurons (reviewed in Horton and Ehlers, 2004; Gonzalez et al., 2018; Kennedy and Hanus, 2019). However, to date, Golgi structures have been identified in dendrites, and not in axons, in the central nervous system (CNS). In peripheral neurons, Golgi structures have been detected in both the dendrites (see below) and also recently in axons (Cornejo et al., 2020). Given the importance of the Golgi apparatus in post-translational modifications and anterograde transport of newly synthesized soluble and membrane proteins, more extensive studies have been carried out on the local secretory systems of dendrites compared with axons and, therefore, this review will focus on the local secretory organelles of dendrites.

The possibility of local protein synthesis and delivery to the dendrite cell surface emerged after the identification of secretory organelles in the dendritic network (reviewed in Valenzuela and Perez, 2015; Britt et al., 2016; Kennedy and Hanus, 2019). A key finding was the identification of small discrete Golgi units in dendrites separated from the main perinuclear Golgi structure (Gardiol et al., 1999; Pierce et al., 2001; Horton and Ehlers, 2003). These dendritic Golgi units are known as Golgi outposts (GOs) (Horton and Ehlers, 2003). Subsequent to their identification, local secretory organelles have been shown to be essential for dendritic development and maintenance in neuronal cell systems.

The identification of local secretory system raises many questions. Can all dendritic membrane proteins, or only a subset, utilize the local trafficking pathway? What are the molecular mechanisms governing this process? Does dysregulated local protein delivery lead to neurological diseases? A key challenge in addressing these questions is to find suitable cell-based models to study membrane trafficking and protein sorting in neurons. Here we review the findings describing local protein trafficking pathways in dendrites, and the role of Golgi structures, obtained from different neuronal systems, point out the strengths and limitations of these models, and emphasize the emerging opportunities of using human neuron systems for elucidation of local protein secretory routes in dendrites.

## Dendritic Secretory Organelles in *Drosophila* Neurons

**FIGURE 1:**
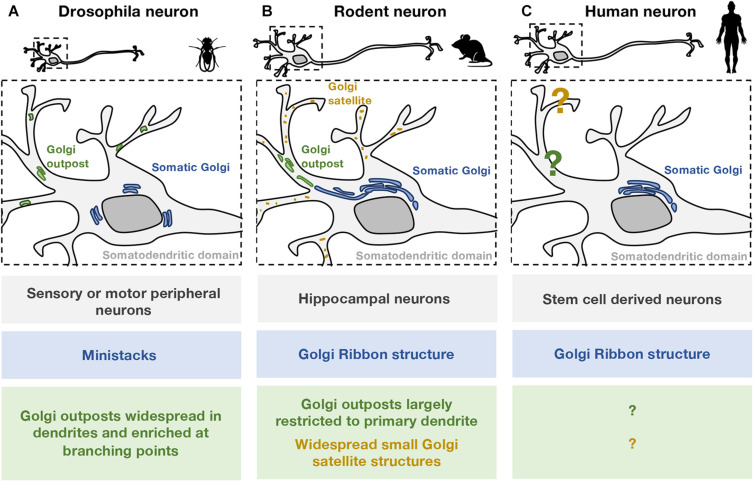
Comparison of somatic and dendritic Golgi structures in different neuronal models. The organization of Golgi structures in **(A)** *Drosophila*, **(B)** rodent, and **(C)** human neurons are illustrated. Zebrafish neurons are not included as the Golgi structures are not well defined. **(A)** In *Drosophila* cell models, most neurons examined are from the peripheral nervous system. In these neurons, the somatic Golgi apparatus (blue) appears as mini-stacks or “ring”-like stacks. GOs (green) are widespread in the dendritic network including the distal dendrites, and are particularly enriched at branching points. Both single- and multi-compartmented Golgi outposts are present in the *Drosophila* dendritic network. **(B)** In rodent models, most neurons examined are cultured embryonic hippocampal neurons. In these neurons, the somatic Golgi apparatus is a Golgi ribbon (blue), and appears to extend into the primary dendrite. Stacked GOs (green) are largely restricted to one primary dendrite and are often found in the proximal region. Smaller, non-stacked Golgi satellite structures (orange) are identified in the dendritic arborisation of rodent neurons. **(C)** In human neurons, the arrangement of secretory organelles, including the Golgi apparatus, is not well defined. A dendritic Golgi in human neurons has yet to be identified. Given the structural differences observed in *Drosophila* **(A)** and rodent **(B)** neurons, conclusions about human neurons should be drawn carefully especially in relation to “Golgi outposts.”

*Drosophila melanogaster* has served as a useful model organism to investigate the formation and function of decentralized secretory organelles in neurons, because of its structural simplicity, short life cycle, easy expansion of progeny, and well-developed genetic manipulation tools (Brand and Perrimon, 1993). In *Drosophila* neurons, both ER and the Golgi structures have been identified within the dendritic extensions (Kondylis and Rabouille, 2009). The ER appears as a network with membrane sheets and tubules abundant throughout the entire neuron (Summerville et al., 2016; Lee et al., 2018). GO structures observed in dendrites appear as small punctate structures by optical microscopy and are scattered throughout the entire dendritic arbor including dendrite tips, and particularly enriched at branching points (Ye et al., 2007; Ori-McKenney et al., 2012) (Figure 1A). In *Drosophila* the Golgi is observed as dispersed mini-stacks in all cells and does not form the higher order ribbon structures found in vertebrates (Kondylis et al., 2001; Kondylis and Rabouille, 2009). In *Drosophila* neurons, the Golgi in the soma or cell body is also found as a set of juxtanuclear ministacks composed of the conventional *cis*, medial and *trans* sub-compartments. In contrast, the *cis*, medial and *trans-*compartments in the dendrite GOs are often not all connected into a single unit (Zhou et al., 2014). The Golgi protein GM130 is a known regulator of the assembly of Golgi stacks (Barr et al., 1998), and GM130 null mutant *Drosophila* display disrupted cisternae stacking of the somatic Golgi and dendritic GOs (Zhou et al., 2014).

The function of dendrite GOs has been directly examined using the *Drosophila* system. Ablation of GO with laser irradiation leads to a reduction in dendritic extension and retraction (Ye et al., 2007), properties which are critical for the regulation of dendritic morphogenesis, indicating that GOs are important for dendrite growth and branching activities. In addition to a potential role in secretory transport, GOs interact with cytoskeletal remodeling and motor proteins. GOs can modulate dendritic organization by serving as microtubule nucleation centers, as they co-localize with the microtubule end binding protein EB1 (Ori-McKenney et al., 2012). By disrupting interactions between the Golgi membranes and the dynein/dynactin motor complex, GOs were redistributed and strikingly, regions with more localized GO displayed a higher degree of dendritic branching (Ye et al., 2007). The localization of GO to the dendrites has been shown to be regulated by a balance of the dynein motor protein with the auto-inhibition of kinesin-1; kinesin-1 mutants which lack auto-inhibition display mis-localization of GO to the axons (Kelliher et al., 2018), indicating the role of motor proteins and the cytoskeleton in precise positioning of the local Golgi structures. The leucine-rich repeat kinase (Lrrk), which localizes to both somatic Golgi and GOs in dendrites, was demonstrated to regulate the movement and directionality of GOs in *Drosophila* neurons via interactions with dynein (Zheng et al., 2008; Lin et al., 2015). A gain of function LRKK2 mutation promoted transport of GOs toward the cell body (retrograde transport) and was associated with suppression of dendritic branching (Lin et al., 2015). Collectively these studies demonstrate that the spatial position of GO within the dendritic network is regulated by the cytoskeleton and associated motor proteins and is essential for dendritic morphology and maintenance.

The *Drosophila* neurons visualized in many of the studies described above used class IV dendritic arborization (da) neurons, which are sensory or motor neurons from the peripheral nervous system (PNS). Hence, these studies may not be representative of neurons from the CNS, particular of the CNS from higher mammals. Although *Drosophila* has provided the opportunity to examine neurons in *in vivo* settings, one caveat of the imaging studies in *Drosophila* is that the third instar larvae have often been used; larval neurons are undergoing development and may not reflect a mature state. The studies on GOs in *Drosophila* are often generalized for all neurons, which may not be appropriate as the Golgi characteristics differ between *Drosophila* and vertebrates (Yano et al., 2005; Kondylis and Rabouille, 2009). Although the presence of the GOs in *Drosophila* neurons clearly indicates the possibility of local secretory traffic within dendrites, the *Drosophila* model has not, to date, been used to obtain direct evidence of cargo trafficking through the local GO and ER organelles (Table 1).

**TABLE 1:**
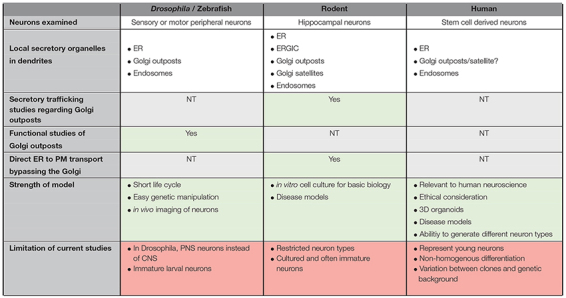
Comparison of organization and trafficking studies associated with the secretory pathway of neurons in different model organisms.

*NT, not tested. Colors provide discrimination of the sections.*

## Role of Golgi in Dendrite Morphogenesis in Zebrafish

Zebrafish (*Danio rerio*) has become a popular model system to study embryogenesis, due to their rapid growth, high numbers of offspring, and ease of genetic manipulation. Also, the transparency of the zebrafish larvae allows *in vivo* neuronal image analysis. Transgenic zebrafish have been used as a model of various neurodegenerative diseases (Tomasiewicz et al., 2002; Kalueff et al., 2014; Nyuzuki et al., 2020). The Golgi of adult zebrafish cells is organized as a ribbon structure in the perinuclear location of most cells (Sepich and Solnica-Krezel, 2016; Saraste and Prydz, 2019). Neurons are an exception and have both a Golgi ribbon and mini-stacks. In addition to the perinuclear Golgi, a fluorescently-tagged Golgi marker identified a Golgi stack within the soma which localized to the base of one neurite during morphogenesis that became the primary dendrite in Purkinje neurons of the zebrafish cerebellar cortex (Tanabe et al., 2010). Furthermore, ongoing secretory trafficking via the Golgi was required for dendrite development. Protein kinase C was identified to play a role in dendrite development and a protein kinase C mutant resulted in altered Golgi localization and aberrant architecture of the Purkinje cells (Tanabe et al., 2010). In other studies, a number of components which regulate Golgi trafficking, including the coat protein COPI (Li et al., 2015) and the tethering complex TRAPPC6B (Marin-Valencia et al., 2018), have been shown to be critical for neuronal development, highlighting the role of the secretory pathway in regulating the establishment of the polarized mature neuron in zebrafish.

## Local Protein Trafficking in Rodent Neurons

Rodent neurons have provided *in vitro* cell culture models to directly examine local protein synthesis and trafficking in dendrites. Similar to *Drosophila* and zebrafish, local secretory organelles have been identified in rodent neurons in both dissociated *in vitro* cell cultures as well as brain slices. The ER spreads throughout the entire neuron as a continuous network of sheets and tubules (Spacek and Harris, 1997; Cooney et al., 2002; Cui-Wang et al., 2012; Wu et al., 2017). In hippocampal neurons from adult rat or mouse brains, EM images show the ER extending as far as the dendritic spines (Cooney et al., 2002; Cui-Wang et al., 2012; Wu et al., 2017). In addition, the smooth ER can invade a subset of dendritic spines, known as the spine apparatus, and is considered to have a role in local transport (Gray and Guillery, 1963; Spacek and Harris, 1997). In cultured rat hippocampal neurons, in addition to the somatic Golgi apparatus, the Golgi was also detected in the dendrites by immunofluorescence using Golgi markers, and by EM, as discrete and independent outpost structures (GOs) (Horton and Ehlers, 2003; Horton et al., 2005; Quassollo et al., 2015; Mikhaylova et al., 2016). In contrast to *Drosophila* neurons where GOs are wide-spread at tips and branching points of dendrites, GOs in rodent neurons are often constrained to the proximal primary dendrite (Horton and Ehlers, 2003; Horton et al., 2005; Quassollo et al., 2015; Mikhaylova et al., 2016) (Figure 1B). In addition to GOs, small dendritic GS structures that do not have the typical cisternal stack organization were also observed in more than 90% of dendrites of rat hippocampal neurons transfected with a Golgi marker containing Golgi-localization sequences from the *trans*-Golgi protein Calneuron-2 (Mikhaylova et al., 2016). In addition to dendritic ER and Golgi structures, other compartments of the secretory pathway have been noted in dendrites of mouse and rat hippocampal neurons, including the intermediate compartment or ERGIC (Pierce et al., 2000, 2001; Hanus et al., 2014), as well as endosomes and transport vesicles (Cooney et al., 2002; Wu et al., 2017).

### Evidence of Dendritic Protein Synthesis and Trafficking in Rodent Neurons

Local protein translation in dendrites of rat neurons has been investigated in some detail (reviewed in Smith et al., 2001; Holt and Schuman, 2013). Abundant mRNA species have been identified in dendrites and dendritic spines encoding membrane receptors, voltage gated ion channels, scaffolds, cytoskeleton, signaling, and transport proteins (Cajigas et al., 2012). Protein translation in dendrites of cultured rat hippocampal neurons has been directly visualized using GFP as a reporter (Aakalu et al., 2001). Subsequently, the synthesis of specific dendritic membrane proteins, the AMPA receptor GluR1, was observed locally in the dendrite (Smith et al., 2005) and the synthesis of GluR1 and GluR2 was detected in dendrites physically isolated and independent of the cell body (Ju et al., 2004).

The trafficking routes of proteins synthesized locally in dendrites have been monitored using techniques to synchronize cargo transport. The thermal-sensitive VSVG mutant model cargo was visualized in ERGIC and GOs after exit from the dendrite ER of cultured rat hippocampal neurons (Horton and Ehlers, 2003; Horton et al., 2005; Hanus et al., 2014); subsequently, the VSV-G cargo was released in transport carriers (Horton et al., 2005; Hanus et al., 2014). The transport of the dendritic cargos, namely, GABA type B metabotropic (GABA_*B*_) receptor, AMPA-type glutamate receptor GluA1, and the adhesion molecule neuroligin 1, have been studied using a reversible dimerization system allowing synchronized release of cargo (Valenzuela et al., 2014; Bowen et al., 2017). In rat hippocampal neurons, GABA_*B*_ receptors were found in both somatic and dendritic ER (Valenzuela et al., 2014) and were transported to both the somatic Golgi and GOs in the dendrites (Valenzuela et al., 2014). GluA1 and neuroligin 1 have also been tracked in rat hippocampal neurons (Bowen et al., 2017). By inclusion of a photo-convertible fluorescent tag, ER cargo in dendrites were shown to traffic predominately to dendritic punctate structures (potentially GOs), whereas somatic ER cargo was transported to the somatic Golgi (Bowen et al., 2017). This finding clearly demonstrates selective trafficking from the dendritic ER to the dendritic GO-like structures. Transport carriers loaded with newly synthesized cargo also co-localized with Rab11, indicating that anterograde cargo transport may occur via the recycling endosomes in dendrites (Bowen et al., 2017).

There is evidence that GOs act as protein sorting stations in dendrites. For example, dendritic GOs have been shown to have a role in selective supply of particular subtypes of glutamate receptors to the plasma membrane. NMDA receptors, which modulate the strength of the synaptic transmission, were observed to accumulate in GOs when Golgi exit was blocked, whereas AMPA receptors, a glutamate receptor which mediates fast synaptic transmission, were retained in the somatic Golgi with disrupted Golgi exit (Jeyifous et al., 2009). Members of a synaptic scaffold protein family, namely, synapse-associated protein-97 (SAP97) and calmodulin-associated serine/threonine kinase (CASK), were shown to be essential for the differential sorting of AMPARs and NMDARs to the somatic and dendritic secretory pathways (Jeyifous et al., 2009). SAP97 has also been shown to associate and regulate the trafficking of the α-secretase, ADAM10, from GOs in dendrites to synaptic membranes, a process modulated by the phosphorylation of SAP97 by protein kinase C (Saraceno et al., 2014). These findings indicate that the dendritic ER and GOs constitute a secretory pathway which is not only spatially separated from the somatic pathway but is also functionally distinct from the conventional secretory pathway.

Analysis of glycosylation of neuronal membrane proteins has indicated that the dendrites have the machinery for Golgi glycosylation (Torre and Steward, 1996). However, the extent of Golgi glycosylation remains an open question as some studies suggest the absence of conventional Golgi processing events. In cultured rat hippocampal neurons, many cell surface glycoproteins have the core high mannose N-glycans synthesized in the ER but lack the typical N-glycans which are generated in the Golgi (Hanus et al., 2016). Brefeldin A treatment, which inhibits transport between the conventional ER and Golgi, has little impact on delivery of nascent cell surface proteins in neurons (Hanus et al., 2016). These findings raise the possibility that the dendritic GOs functionally differs from the somatic Golgi and/or there are transport pathways from the ER/ERGIC to the dendritic cell surface independent of the Golgi.

### Synaptic Control of Protein Translation and Transport in Dendrites

A number of investigations have analyzed local protein synthesis and transport arising from synaptic activity. In cultured rat hippocampal neurons, excitatory synaptic stimulation enhanced synthesis of AMPA receptors locally in the dendrites (Smith et al., 2005), rather than the central cell body (Ju et al., 2004; Smith et al., 2005; Sutton et al., 2006). Moreover, local protein synthesis was found to be essential to synaptic plasticity in response to neurotrophic stimuli (Kang and Schuman, 1996).

Membrane transport of nascent proteins to their functional destinations has also been reported to be under synaptic control. In cultured rat hippocampal neurons, increased neuronal firing reduced mobility of VSVG-loaded transport carriers in the dendrites following ER release (Hanus et al., 2014), while decreased synaptic activity promoted the mobility of transport carriers over long distances (Hanus et al., 2014). The authors propose that the confinement of transport carriers within local sites may provide focused cargo trafficking to specific dendritic membrane domains associated with synaptic signaling events.

Based on these studies, dendritic proteins are synthesized locally upon synaptic stimulation presumably to meet the requirement of fast protein supply at remote dendritic sites particularly the spine plasma membrane, to perform synaptic transmission. However, the details of the local transport pathways to the dendritic cell surface remain unclear and various scenarios have been suggested (Horton et al., 2005; Kennedy et al., 2010; Hanus et al., 2014; Bowen et al., 2017). Are cargoes transported directly to the surface after ER-exit, or via the ERGIC, GOs, or Rab11-positive endosomes? Do different receptors and ion channels share the same pathway(s) or are they transported by different routes with each route defined by specific transport machinery? These are key questions worthy of further investigation.

Cultured rodent neurons provide a valuable system to analyze local secretory cargo transport. Nonetheless, and in contrast to *Drosophila* and zebrafish, functional studies of the GOs in the rodent systems have yet to be carried out. In addition, most of the studies performed using rodents have focused on one specific type of neuron, namely hippocampal neurons, which may not be representative of other types of neurons in the CNS. Furthermore, trafficking assays carried out with monolayers of neurons in cell culture may not reflect accurately the regulation of trafficking of neurons within the intact, mature brain environment.

## Membrane Protein Trafficking in Human Neurons

Rodent neurons may not replicate all aspects of human neuron biology; however, the application of human neurons in mapping transport pathways has significant challenges. There have been a limited number of studies on primary human neurons obtained from deceased individuals (LeBlanc, 1995; LeBlanc et al., 1997; Sivananthan et al., 2010). Immortalized cell lines from neuroblastoma lines are frequently used as a cell model to primary human neurons (reviewed in Gordon et al., 2013). Although immortalized cell lines can resemble neuron morphology with extending neurites, they do not display the functionality of primary neuronal cultures with active synaptic transmission (LePage et al., 2005).

The advancement in human stem cell technology has opened up exciting possibilities for the study of human neurons. With the development of induced pluripotent stem cells (iPSCs) (Takahashi and Yamanaka, 2006), iPSCs from patients or healthy individuals can be differentiated into different cell types, including neurons (Figure 1C). Over the last decade, human iPSC databases have become available, for example, DIAN (Karch et al., 2018), which allows human iPSC-derived neurons to be widely applied to neurological disease modeling (reviewed in De Filippis et al., 2017; Li et al., 2018), with established protocols to differentiate different types of neurons both in 2D and 3D culture (Karch et al., 2018; Costamagna et al., 2019; Ooi et al., 2020).

To date, only very limited studies have explored membrane trafficking pathways using human iPSC derived neurons. Notably, intracellular organelles have been identified in human iPSC derived neurons, and axonal transport of endosomes and mitochondria have been recently reported (Boecker et al., 2020). Of relevance is that the extension of the somatic Golgi into the neurite has been observed from a patient iPSC derived neurons (Lemonnier et al., 2011). However, the relationship between the extensions of Golgi structure from the soma of iPSC derived neurons and the distinct GOs observed in other neuronal cell systems remains unclear; no systematic study has been performed mapping local secretory pathways in human neurons.

Perturbations in protein sorting and Golgi abnormalities are linked to a variety of neurological disorders and diseases. Human iPSC-derived neurons from patients therefore have considerable potential to identify the underlying molecular mechanisms. For instance, complex hereditary spastic paraplegia in children results in loss of function of the TGN AP-4 cargo adaptor (Behne et al., 2020). Analysis of AP-4 deficient human iPSC-derived neurons from patients showed reduced neural outgrowth and branching, indicating that Golgi transport is affected. Dysfunctional membrane trafficking contributes to a number of neurogenerative disease (reviewed in Neefjes and van der Kant, 2014; Kiral et al., 2018), for example, Alzheimer’s disease where altered membrane trafficking results in enhanced Aβ production (Tan and Gleeson, 2019). Neurons derived from iPSC carrying familial Alzheimer’s disease mutants exhibit Alzheimer’s disease related phenotype including increase amyloid β and phosphorylated tau (Ochalek et al., 2017). There is considerable potential for mapping transport pathways in human primary neurons using iPSC derived neurons and it is likely there will be major advances in this area in the next few years.

## Summary and Conclusion

It is clear that local protein transport machinery at remote sites away from the central cell body is important in facilitating efficient transport of nascent membrane proteins to their functional sub-domains in response to synaptic stimuli. Different model organisms have been utilized to define different aspects of local secretory trafficking in dendrites. Although there is general agreement of local transport in the different models, the organization of neuronal secretory pathways appears to differ across species. For instance, in *Drosophila* neurons, GOs are scattered stations in the entire dendritic network, while in rodent neurons, GOs often extend from the central Golgi into one proximal principal dendrite, but with smaller GSs widely distributed throughout the dendritic network. GOs have been shown to be required for dendrite development in *Drosophila* and zebrafish. However, the pathways for the positioning of the GOs and other organelles of the secretory and endocytic pathways in dendrites are unknown. The application of EM tomography during differentiation of immature neurons into mature neurons would be very informative. Notably the application of EM tomography and live imaging has recently identified a novel high mobile ribosome associated vesicle (RAV) in the dendrites of rat cortical neurons, which have a potential role in local translation (Carter et al., 2020), and highlighting the importance of sophisticated imaging approaches to reveal the local secretory processes.

As there are differences between the systems studied (Figure 1), extrapolation between the different models should be done with caution. Functional studies on GOs in *Drosophila* neurons are often generalized for GOs of other models, which may not be appropriate (Yano et al., 2005; Kondylis and Rabouille, 2009). Table 1 summarizes some of the features of the different models and the aspects of anterograde trafficking that have been examined thus far. As mentioned, in *Drosophila* the Golgi does not adopt the ribbon structures that are found in vertebrates (Kondylis and Rabouille, 2009; Gosavi and Gleeson, 2017). Therefore, vertebrates must have a distinct mechanism for dissociation of individual Golgi stacks from the ribbon structures during neurogenesis. In addition, it has been shown in *Drosophila* imaginal disc cells that the Golgi stacks are heterogeneous and distinct subpopulations have been identified which differ in their profile of glycosylation enzymes (Yano et al., 2005). This finding raises the question whether the GOs in mammalian neurons are functionally identical or whether there may also be subsets of Golgi mini-stacks which differ functionally.

In addition to the transport of newly synthesized proteins in the secretory pathway, recycling of synaptic membrane proteins is also important in the regulation of synaptic function (Groc and Choquet, 2020). The TGN is at the cross roads for receiving and recycling internalized cell surface membrane proteins (Tang, 2008; Lieu and Gleeson, 2011), and GOs could also contribute to these recycling events.

Regardless of the differences across species, the emerging theme is one where secretory transport machinery is tailored in neurons to meet requirements of protein supply for synaptic transmission at sites distal from the cell body. The presence of distinct functional secretory pathways in the soma and dendrites provides the capacity to sort and independently transport the plethora of synaptic membrane proteins, and to provide a fast localized response when required. For example, a subset of nascent synaptic proteins, such as ion channels and receptors, may use the local trafficking pathway to fine tune synaptic responses, while other proteins that are not required for the activity-dependent modulation of synaptic transmission might use the slow, central machinery. More systematic studies need be done to elucidate and quantitate the use of the two secretory pathways, for both protein synthesis and for sorting, by different subsets of neuronal proteins. GOs, serving as a potential local sorting and maturation stations, could play a role in regulating local protein transport to defined plasma membrane domains. It is also likely that novel molecular mechanisms which regulate the transport processes in these secretory pathways in neurons will be revealed as the pathways are further defined.

There are many gaps in the understanding of anterograde transport in human neurons. The spatial distribution of ER/Golgi/endosomes, and the anterograde trafficking pathways, in dendrites of healthy human neurons is unknown. Whereas previous studies have used rodent and insect neurons to analyze membrane trafficking, there is now the opportunity with iPSC to define the pathways in human neurons using sophisticated new technologies to track the itineraries of newly synthesized membrane cargo, such as the RUSH system (Boncompain et al., 2012; Abraham et al., 2016), coupled with super-resolution optical microscopy and 3D EM.

The concept of local protein synthesis and transport needs to be taken into consideration when investigating the molecular basis of neurological diseases. Over the past 15 years perturbations in protein sorting and membrane trafficking have been implicated in a wide range of neurodegenerative diseases, and there have been numerous studies reporting “fragmentation” of the Golgi associated with neurological disorders (Makhoul et al., 2019). However, there is little information on the intracellular location of the trafficking defects in human neurons. An important question yet to be addressed is whether abnormalities of the Golgi structure include perturbations of dendritic GOs and local dendritic trafficking. iPSC-derived neurons from patients with neurological diseases associated with defects in post-Golgi trafficking represent a powerful tool to understand the molecular and cellular processes in human neurons that contribute to the pathology of various diseases and to screen potential new treatments.

## Author Contributions

JW, LF, and PG planned the review and edited the final manuscript. JW wrote the first draft of the review and designed the figure. LF and PG revised the manuscript. All authors contributed to the article and approved the submitted version.

## Conflict of Interest

The authors declare that the research was conducted in the absence of any commercial or financial relationships that could be construed as a potential conflict of interest.

## Abbreviations

CNS: central nervous system
EM: electron microscopy
ER: endoplasmic reticulum
ERGIC: ER-Golgi intermediate compartment
GOs: Golgi outposts
GSs: Golgi satellites
iPSCs: induced pluripotent stem cells
PNS: peripheral nervous system
TGN: *trans*-Golgi network.
